# Latent-space embedding of expression data identifies gene signatures from sputum samples of asthmatic patients

**DOI:** 10.1186/s12859-020-03785-y

**Published:** 2020-10-15

**Authors:** Shaoke Lou, Tianxiao Li, Daniel Spakowicz, Xiting Yan, Geoffrey Lowell Chupp, Mark Gerstein

**Affiliations:** 1 Program in Computational Biology and Bioinformatics, Yale University, New Haven, CT 06520 USA; 2 Department of Molecular Biophysics and Biochemistry, Yale University, New Haven, CT 06520 USA; 3 Division of Medical Oncology, The Ohio State University, Columbus, OH 43210 USA; 4 Pulmonary and Critical Care, Yale School of Medicine, New Haven, CT 06520 USA

**Keywords:** Asthma, Asthma subtypes, Denoising autoencoder, Biomarker, Non-invasive

## Abstract

**Background:**

The pathogenesis of asthma is a complex process involving multiple genes and pathways. Identifying biomarkers from asthma datasets, especially those that include heterogeneous subpopulations, is challenging. Potentially, autoencoders provide ideal frameworks for such tasks as they can embed complex, noisy high-dimensional gene expression data into a low-dimensional latent space in an unsupervised fashion, enabling us to extract distinguishing features from expression data.

**Results:**

Here, we developed a framework combining a denoising autoencoder and a supervised learning classifier to identify gene signatures related to asthma severity. Using the trained autoencoder with 50 hidden units, we found that hierarchical clustering on the low-dimensional embedding corresponds well with previously defined and clinically relevant clusters of patients. Moreover, each hidden unit has contributions from each of the genes, and pathway analysis of these contributions shows that the hidden units are significantly enriched in known asthma-related pathways. We then used genes that contribute most to the hidden units to develop a secondary random-forest classifier for directly predicting asthma severity. The feature importance metric from this classifier identified a signature based on 50 key genes, which are associated with severity. Furthermore, we can use these key genes to successfully estimate FEV1/FVC ratios across patients, via support-vector-machine regression.

**Conclusion:**

We found that the denoising autoencoder framework can extract meaningful patterns corresponding to functional gene groups and patient clusters from the gene expression of asthma patients.

## Background

Asthma is a common chronic disease of the airways. According to a medical expenditure survey in the United States from 2008 to 2013, asthma has a prevalence of 4.8% and imposes significant economic burden, including costs due to missed work and school, medication and mortality [1].

Asthma is recognized as a heterogeneous and complex disease involving many biological pathways [2]. Among asthma patients identified as severe, subpopulations with diverse pathogenicity may exist that respond differently to medications [3]. Thus, identifying distinct subgroups of asthma patients is crucial for personalized medical decisions and management. Several researchers have investigated aspects of asthma heterogeneity and tried to identify subgroups based on different types of indicators. Simpson et al. [4] categorized asthma patients into four subtypes, eosinophilic, neutrophilic, mixed granulocytic and paucigranulocytic, based on the count and components of white blood cells in induced sputum [5]. The Severe Asthma Research Program performed hierarchical clustering on phenotypic measures of 856 patients and revealed five groups with distinct phenotypic features [6]. Yan et al. [7] identified three transcriptomic endotypes of asthma (TEAs) using unsupervised clustering on gene expression of induced sputum of asthma patients, demonstrating the predictive potential of molecular profiles on disease phenotypes. Each of these studies tried to interpret the identified subgroups by investigating how they associate with disease phenotypes, but did not explicitly evaluate their association with disease severity. Thus, we still lack a stringent set of indicators of severe phenotypes. As many asthma subgroups contain a non-trivial proportion of severe patients, work is needed to further characterize specific genes or pathways that lead to more severe phenotypes within each patient subgroup.

Scientists have extensively used transcriptome profiling to study human diseases at a molecular level. Gene transcripts that show significant differential expression and structural aberrations associated with disease phenotypes provide promising markers of clinical significance [8]. Easily obtained non-invasive biospecimens are useful sources of markers with high potential for convenient and efficient clinical applications [7]. Hekking et al. identified differentially expressed gene and pathway signatures for adult- and childhood-onset severe asthma from transcriptomes of brushings and sputum [9].

The pathogenicity of asthma involves complex and non-linear interactions between several biological pathways [10]. Thus, higher-order, non-linear features will be necessary to capture the intrinsic structure of gene expression data. Researchers can apply non-linear generative models in order to obtain more stable representations of the data for robust feature extraction. For example, a denoising autoencoder attempts to reconstruct the original data from a randomly corrupted input, and the resulting model can potentially map the high-dimensional input data to lower-dimensional representations that are robust to small noise in the input [11]. This framework is therefore suitable for extracting useful features in noisy and high-dimensional transcriptome data. As an example, Tan et al. applied this method to breast cancer gene expression data and identified features that are related to the prognosis of patient survival [12].

To reveal the intrinsic structure and to extract predictive features from heterogeneous transcriptome data, we propose a framework called dAsthma that uses a denoising autoencoder to generate robust and non-linear representations with clinical relevance. We argue that (1) this simple structure can retrieve components that have biological relevance and are explainable, (2) the hidden units produce clearer patterns than the raw data to categorize patients into heterogeneous groups, and (3) components of the clinically relevant hidden units may contain genes that are functionally associated with the pathogenesis of asthma, serving as potential sources for biomarker discovery.

## Results

### Training the denoising autoencoder

**Fig. 1:**
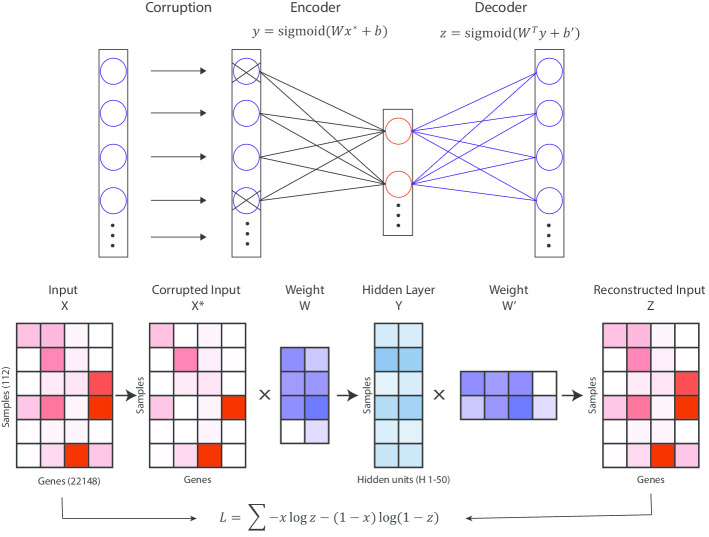
Denoising autoencoder model architecture

We used a one-layer denoising autoencoder model comprised of an encoder and a decoder (Fig. 1). The encoder embeds the original input data into a lower-dimensional space, the hidden layer, and the decoder reconstructs the input from the values of the hidden layer. We tuned the parameters of the model using cross-validation by training on 90% of the randomly selected sample and testing on the remaining 10% and repeated this process 10 times. Both the training and testing loss showed proper convergence (Additional 1: Fig S1, S2), indicating that we largely avoided overfitting. We then projected the input data from all non-control input samples to the embedding space of the trained model, obtaining a set of 50-dimensional vectors.

### Hidden units associate with TEA clusters

**Fig. 2:**
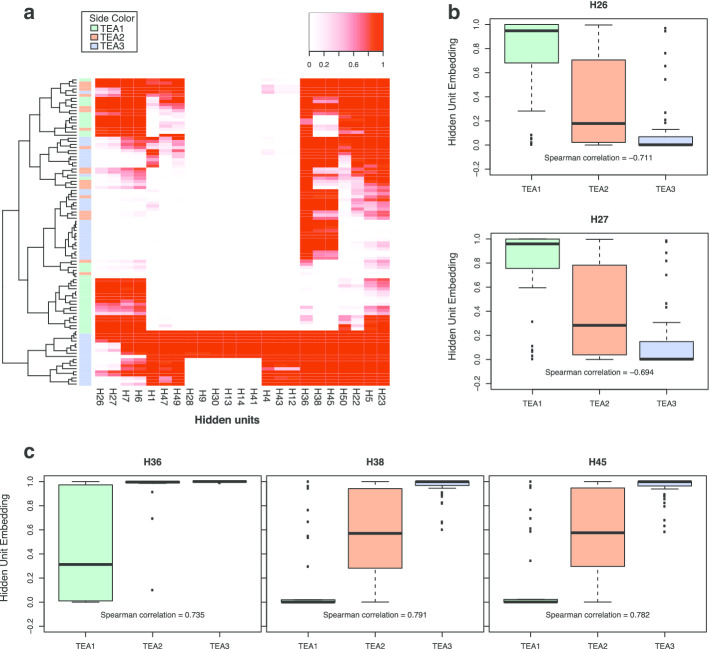
Identification of clinically relevant hidden units. **a** Heatmap of hidden layer embeddings. The sidebar indicates the TEA cluster assignment of the sample. **b** Hidden units that are negatively correlated with the TEA cluster label (H26, H27). **c** Hidden units that are positively correlated with the TEA cluster label (H36, H38, H45)

By encoding the original data into the hidden vector space, the model produced a sparse embedding space; moreover, we could observe distinct patterns related to clinical traits (Fig. 2a). Some hidden units showed approximately complementary behaviors (e.g., H26 and H38), indicating their distinct relevance to key molecular mechanisms and clinical subgroups of patients.

To better interpret the learned patterns, we evaluated the embeddings of all input data for their correlations with identified clinical traits. We first removed hidden units with variance < 0.001. Using the embeddings of the highly variable hidden units, the samples formed several small clusters corresponding to the previously defined TEA clusters, with high homogeneity (Fig. 2a). We found that TEA1 contained a larger proportion of severe patients than TEA3 (Additional 1: Fig S4). TEA2 was relatively harder to distinguish because it was somewhat of an intermediate between TEA1 and TEA3, as some of the TEA2 samples were spread across several clusters.

Generally, the values of hidden units showed gradual monotonic changes, either increasing or decreasing, from TEA1 to TEA3 (Additional 1: Fig S5), indicating associations of the hidden units with clinical traits of the samples. We selected five hidden units (H26, H27, H36, H38, H45) that were significantly correlated with TEA cluster labels (Spearman correlation > 0.65), namely *Hsig*. Based on the performance of these five hidden units, we further categorized them into two major classes: one that was negatively correlated with TEA cluster labels (H26, H27; Fig. 2b) and one that was positively correlated (H36, H38, H45; Fig. 2c).

### Annotation of relevant hidden units

To further understand the biological significance of the hidden units, we tried to associate them with functional enrichment based on the weights of the encoder network that mapped the input to the hidden unit space. This weight represents the contribution of the gene to the value of the hidden unit and can be considered the component of the hidden unit. Thus, we could infer biological significance of the hidden units from the weights of the encoder.

**Fig. 3:**
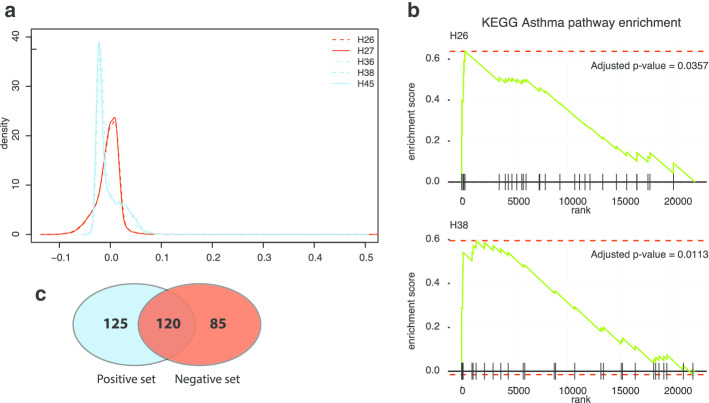
Annotation of hidden units. **a** Distribution of encoder weights of *Hsig*. The positive and negative sets show very different distribution patterns. **b** Gene set enrichment of the asthma pathway from KEGG (KEGG_ASTHMA) for H26 and H38. **c** Intersection between top weighted 200 genes of the positively (H26, H27) and negatively (H36, H38, H45) related hidden units (duplicates removed)

The weight distribution of the encoder layer showed similar patterns for hidden units belonging to the same set (Fig. 3a). The negative set showed a nearly symmetric distribution with a slight negative skew, whereas the positive set showed a highly positively skewed shape. Using encoder weights for all 22,148 genes as the ranking score, we performed gene set enrichment analysis using Kyoto Encyclopedia of Genes and Genomes (KEGG) pathway gene sets to obtain functional enrichments of *Hsig*. Similar to the weight distribution, hidden units from the same set showed strong resemblance with respect to enrichment of functional terms (Additional 1: Fig S6). Notably, many of the enriched functional terms were related to molecular functions and signaling pathways associated with disease pathogenesis and autoimmune response, whereas deficient terms included “gene expression machinery” and “metabolism”. Specifically, the enrichment of the term “asthma” showed high significance in all five hidden units (Fig. 3b, Additional 1: Fig S7).

We then extracted the top-weighted genes of *Hsig* as potential clinical markers for downstream analysis. For each hidden unit, we extracted the top 200 genes with the highest weights; we removed ribosome-related genes from the analysis to prevent ribosome functional roles from dominating the selected gene set. Concordant with previous observations, the gene sets for hidden units belonging to the same class were highly similar. By merging the top-weighted genes from all five hidden units, we obtained a list of 330 genes. We identified several genes that showed distinct differential patterns of weights between the positive and negative set (Fig. 3c). Gene ontology (GO) analysis revealed an enrichment of disease-related terms like “antigen processing and presentation”, “immune response” and “interferon-gamma signaling pathway” (Additional 1: Fig S8).

### Top-weighted genes associate with asthma severity

In order to assess the clinical relevance of the learned model, we investigated the association between hidden unit values and clinical data. Generally, the performance of hidden units showed distinct correlations with various clinical traits (Additional 1: Fig S9). We then tested the predictive performance of some clinical traits using the embedding values of *Hsig* and the expression of the combined list of their top-weighted genes.

**Fig. 4:**
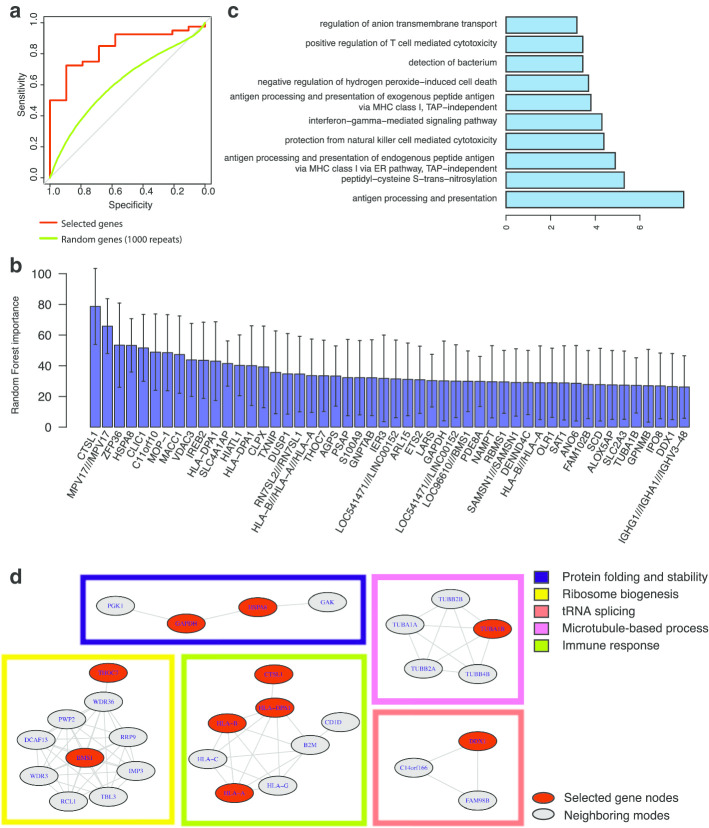
Prediction of asthma severity and feature selection. **a** AUROC of the prediction of asthma severity using selected genes compared to randomly selected genes. **b** Importance of the selected genes. **c** GO term enrichment of the selected genes. **d** Selected genes in the context of a PPI network

We first associate the value of all hidden units, *Hsig* and the top-weighted genes with the asthma severity levels of the patients. We only used samples labeled as “mild” or “severe” for the classification analysis. Given each training dataset, we trained a random forest classifier and assessed the predictive accuracy on the test data, represented by the area under the receiver operating characteristic curve (AUROC) value (Fig. 4a).

From the top-weighted genes, we then performed feature selection to obtain the most relevant genes. We considered 50 genes with the top average importance as the most relevant to the prediction of severity (Fig. 4b, Additional 1: Table S1). Expression of these genes reached an average AUROC of 0.8066 (average AUROC is 0.6221 for randomly selected 50 genes from all expressed genes) in predicting severity level. This indicates the top weighted genes are related to severity and the latent patterns unsupervised learned by the autoencoder are biological relevant.

We also evaluate the performance of differentially expressed genes (DEGs) from severe versus mild patient group (AUROC: 0.9318, see Methods for details) (Additional 1: Fig S10). There is no overlapping between our selected genes with DEGs, but network analysis showed that our selected genes have significantly higher interconnectivity and higher centrality in the network (Wilcoxon test, p-value = 0.003) compared to the DEGs (Additional 1: Fig S11). This is not surprising since the severity related DEGs is specifically selected to be discriminative. In contrast, the top-weighted genes are defined from the most representative patterns among all the samples (including control, mild, moderate and severe groups) learned by unsupervised learning. They are not necessarily associated with severity, but can still achieve high predictive performance, though not as high as specific DEGs. Also, they play more central roles through network interactions. This further indicates our framework can capture the biological relevant information.

Our list of selected genes included genes related to autoimmune responses, such as antigen processing and presentation, T-cell toxicity, interferon signaling and cell adhesion (Fig. 4c). In the context of a protein–protein interaction network, we identified genes residing in various functional modules (Fig. 4d). Specifically, several genes, such as human leukocyte antigen genes and cathepsin L1, belonged to a module related to immune response. In addition, the list included genes with potential relevance to asthma pathogenesis, such as immunoglobulin heavy chain (IGHG1, IGHA1 and IGHV3-48), inflammation (S100A9) [13] and iron response (IREB2) [14, 15] genes.

### Prediction of clinical measurements

To evaluate an individual’s lung function, clinicians use the ratio of the volume forcefully exhaled in one second versus the maximum volume of a forceful exhale (i.e., the FEV1/FVC ratio) [16]. A significantly reduced FEV1/FVC ratio is a sign of airflow obstruction, and is considered a criterion for asthma severity.

**Fig. 5:**
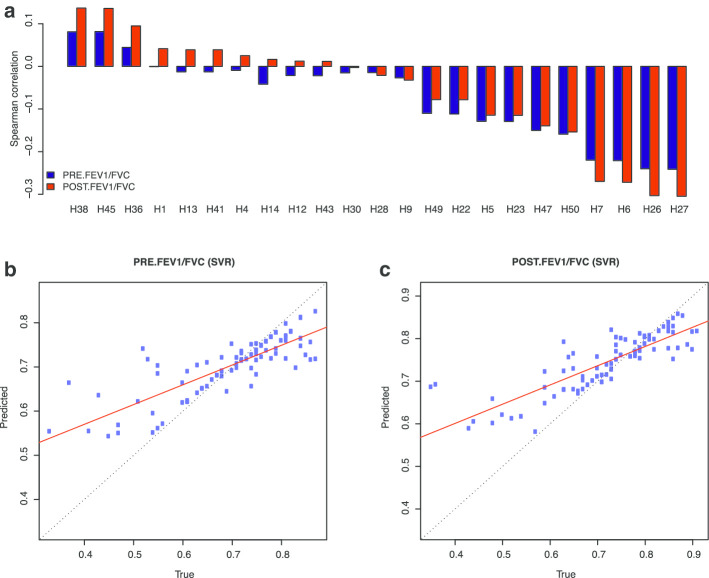
Prediction of FEV1/FVC ratio. **a** Spearman correlation between hidden unit values and pre-/post-treatment FEV1/FVC. **b** Plot of predicted value versus true value of pre-treatment FEV1/FVC using support vector machine regression with selected gene expression. **c** Plot of predicted value versus true value of post-treatment FEV1/FVC using support vector machine regression with selected gene expression

We found that the hidden units in *Hsig* generally showed a higher absolute value of correlation with the FEV1/FVC ratio, in both the positively and negatively correlated subsets, compared to other hidden units, indicating clinical relevance of these hidden units (Fig. 5a).

Finally, we tried to predict the pre- and post-treatment FEV1/FVC ratios with the value of hidden units and expression of their top-weighted genes using a support vector regression (SVR) and least absolute shrinkage and selection operator (LASSO) (Fig. 5b, c, Additional 1: Table S2, S3 and Fig S12, S13). The selected genes achieved the highest predictive performance, in terms of both mean squared error and explained variance. Together, these results suggest that the selected hidden units and genes are clinically relevant and could be used as markers for asthma severity.

## Discussion

Research suggests that asthma is a heterogeneous disease, as its pathogenicity may largely depend on a patient’s individual genetic variability [17]. For several previously proposed subsetting methods of the asthma population, many of the resulting subpopulations contain a non-trivial proportion of severe cases [7]. This indicates that severe asthma may be the result of multiple subtype-specific mechanisms. Thus, diagnosis from disease phenotypes solely may not provide sufficient information for personalized treatment. To identify the molecular regulatory mechanisms associated with asthma severity, we developed a framework called dAsthma using a denoising autoencoder model trained on gene expression profiles of sputum from asthma patients. The model can map high-dimensional expression data to a lower-dimensional latent vector space composed of 50 hidden units, and cluster the patients into clinically relevant subgroups using the embeddings in the latent space. We then investigated how the hidden units were associated with biological pathways and clinical traits. From the most relevant hidden units, we discovered a set of 50 genes whose expression profiles combined could predict asthma severity with high accuracy.

The dAsthma framework learns a more robust representation of the data by adding random noise to the input data. It looks for definitive features that account for the variations among the dataset. Compared with raw gene expression data, which produce less distinguishable patterns in clustering analysis, the hidden unit values learned by the dAsthma model can identify distinct clusters that partly overlap with previously identified patient groups out of the noisy data. We note that these clusters are generated in an unsupervised fashion, that is from mere gene expression data with no external information provided. We tuned the autoencoder model of our framework to make it more robust. We selected an optimal learning and corruption rate by performing a grid search of hyper-parameters. The dataset that we used to train our model was relatively small; this may limit the ability of the autoencoder to identify underlying patterns of the data due to lack of information, and could introduce an additional risk of overfitting. We tested our model using cross-validation and found good convergence of both training and test loss. We selected the model architecture (i.e., the number of hidden nodes) based on the following considerations: As a smaller number of hidden units may fail to capture some subtle structures among the dataset, we used a relatively larger and presumably redundant number (50 hiddenunits), allowing us to retain as much useful information as possible. We then filtered the 50 learned hidden units for conciseness and specificity. Among these hidden units, some showed almost uniform values across all samples; these were of less interest and were discarded. In addition, some highly variable hidden units showed similar performance and components. We collapsed these highly correlated hidden units for downstream analysis.

Neural network-like models become less interpretable as they grow deeper and more complex. Especially when applied to biological data, associating a learned model with the underlying mechanisms of molecular functions and disease pathology is challenging. Our dAsthma framework uses a simple, low-complexity structure to achieve better interpretability. The learned patterns can be interpreted from two perspectives. The first is to study the enrichment of biological pathways of the hidden units based on their components (i.e., genes weighted by their contributions to the hidden units). The second is to associate the patterns with external information about clinical measurements, such as asthma severity. These analyses showed that the hidden representations learned by the denoising autoencoder could bridge gene expression and clinical traits. Overall, the hidden units can be recognized as “gene modules” that represent key biological pathways in the pathogenicity of asthma, which lead to various disease phenotypes.

The definition of asthma severity is mainly based on phenotypic traits and may vary across studies. The top severity-associated genes selected from the components of the most clinically relevant hidden units potentially could be used to characterize asthma severity for different subtypes, as the denoising autoencoder in dAsthma tends to identify features that account for variations among the asthma patient population. We also showed that our selected genes, though showing lower predictive ability, generally have more central biological roles in the interaction network than severity related DEGs. Previous work by de la Fuente [18] highlighted that key regulators that significantly alter pivotal biological processes in diseases are not always found in the most differentially expressed genes. Thus, integrating regulatory or co-functioning information beyond mere differential expression would facilitate the identification of disease-causing genes and pathways. As our autoencoder-based framework could detect such information in an unsupervised fashion, we believe that further exploration of these models on biological data would facilitate the understanding of the function of complex regulatory behaviors especially in human diseases.

## Conclusions

We have demonstrated the strength and application of our dAsthma framework, which makes use of a denoising autoencoder for extracting clinically relevant patterns in an unsupervised fashion. From the patterns, we demonstrate a rational way to select potential biological relevant genes from the noisy gene expression data of sputum of asthma patients. Compared to straightforward differential expression analysis, our method identifies gene signatures with significant higher centrality, which tend to play more pivot role in the biological network.

## Methods

### Data

The raw expression data was provided by Yan [7]. After quantile normalization, all expression data was scaled to [0,1] in a sample-wise fashion by the min–max method, i.e., $\tilde{x} = \frac{{x - {\min}\left( x \right)}}{{\max \left( x \right) - {\min}\left( x \right)}}$, for each sample.

### Implementation and training of the denoising autoencoder

The denoising autoencoder is comprised of two symmetric neural networks: an encoder network and a decoder network. The encoder network first maps a corrupted input $X^{*}$, i.e.,

$$y = sigmoid\left( {Wx^{*} + b} \right)$$

The decoder network then tries to produce a reconstructed input $Z$ from the hidden vector space that resembles the original input as much as possible:

$$z = sigmoid\left( {W^{T} y + b^{\prime}} \right)$$

Specifically, we constrain the weights of the decoder network to be the transpose of that of the encoder. The loss function is cross-entropy loss:

$$L = \mathop \sum \limits_{i} - x_{i} logz_{i} - \left( {1 - x_{i} } \right) log\left( {1 - z_{i} } \right)$$

Then, the derivative can be calculated as follows:

$$\begin{aligned} \frac{\partial L}{{\partial W_{ij} }} & = \left( {z_{j} - x_{j} } \right)y_{i} + \left[ {\mathop \sum \limits_{k} \left( {z_{k} - x_{k} } \right)W_{ik} } \right]y_{i} \left( {1 - y_{i} } \right)x_{j}^{*} \\ \frac{\partial L}{{\partial b_{i} }} & = \left[ {\mathop \sum \limits_{k} \left( {z_{k} - x_{k} } \right)W_{ik} } \right]y_{i} \left( {1 - y_{i} } \right) \\ \frac{\partial L}{{\partial b_{j} ^{\prime}}} & = z_{j} - x_{j} \\ \end{aligned}$$

Weights are shared between the encoder and the decoder (i.e., the weight matrix of the decoder is a transposition of that of the encoder). The output layers of the encoder and decoder are activated with a sigmoid function.

We tuned the parameters of the model using different values. In particular, we tested the hidden nodes from 10 to 70, and epochs with 30, 50, 100 and 150. Finally, as a trade-off between the performance and redundancy of the hidden nodes (Additional 1: Fig S3), we used a model with 50 hidden units, 100 epochs, a learning rate of 0.1 and a corruption rate of 0.001, to minimize the cross-entropy loss. After tuning the parameters, the model was evaluated with random training and test data of the original data set for 10 times: the model was first trained using a randomly selected 90% of the samples, and then tested on the remaining 10%. Cross-validation on the testing set between the true and predicted value was used for the evaluation.

### Gene set enrichment analysis

We used the fgsea function (from the R package fgsea) [19] to perform gene set enrichment analysis. The fgsea function expects an input of statistic array for genes in a gene list of interest, as a measurement of the relevance of the genes to a desired phenotype. In our scenario, for each hidden unit we regard the learned weights of the input features (genes) as the statistic array. We used the curated KEGG gene set from MSigDB v6.2 (downloaded from https://software.broadinstitute.org/gsea/downloads.jsp) for the analysis. We ran the analysis for 1000 permutations. The output was visualized as a plot of enrichment scores against genes ranked by statistical values.

### Prediction of clinical traits

Generally, the prediction of severity (also, the pre- and post-treatment FEV1/FVC ratios) was evaluated using four-fold cross-validation. The data was randomly split into four equal parts. For each part, the target value was predicted using a model trained with the other three parts. The predicted values of the four parts were then concatenated and compared to the true values.

For prediction of severity, only samples labeled as “mild” and “severe” were used for the analysis. We performed a random forest (using the R package caret) on the training data with default parameter settings. Altering the parameters (i.e., the number of trees and number of features for splitting the nodes) did not significantly impact the results (data not shown). The AUROC reported is the average over ten repeats of four-fold cross-validation. We also compared the result with randomly sampled 50 genes and averaged over 1,000 random samplings.

For prediction of the pre- and post-treatment FEV1/FVC ratio, both support vector regression (SVR) using the R package e1071 and LASSO (using the R package glmnet) were used. The predictive power of the trained model was assessed by calculating the Pearson correlation between the predicted values and true values.

### Feature selection

An initial gene set was generated by merging the top-weighted genes for the five most clinically relevant hidden units. The merged gene list, containing 330 genes, was used as input to a random forest classifier to predict the severity label (“mild” or “severe”), with all samples used. For a random forest regression model, the importance of the input variables was calculated as follows: for each tree, the out-of-bag mean**-**squared error was computed before and after randomly permuting a variable. The importance of the variable was defined as the average difference of the out-of-bag mean**-**squared error before/after permutation over all trees.

The importance of the features in the learned model was then extracted from the learned model. We used 50 genes with the highest importance averaged over 50 trials as the selected genes for downstream analysis.

### Differential expression analysis

DEGs related to severity were selected with a linear model against mild/severe labels using package limma [20]. We used genes with adjusted p-value < 0.1 as significant DEGs, resulting in a list of 24 genes.

### Network analysis

Proteins corresponding to the selected genes are provided via STRING (https://string-db.org/). For microarray probes targeting multiple proteins, all of the corresponding proteins are included. To visualize the role of these proteins in the context of a protein–protein interaction (PPI) network, we also included nodes of first-shell interactions (colored in grey in Fig. 4d) with the query proteins (colored in red in Fig. 4d). Only experimentally validated and database-curated interactions are included.

## Supplementary information

**Additional file 1.** Supplementary Information for the analysis. Table S1–S3 and Fig S1–S13 are included in the file.

## Data Availability

All data generated or analysed during this study are included in this published article: Yan X, Chu JH, Gomez J, Koenigs M, Holm C, He X, et al. Noninvasive analysis of the sputum transcriptome discriminates clinical phenotypes of asthma. Am J Respir Crit Care Med. 2015;191(10):1116–25. All codes are available at: https://github.com/gersteinlab/dAsthma

## Abbreviations

FEV1/FVC: Forced expiratory volume in 1 s/ forced vital capacity
TEA: Transcriptomic endotype of asthma
KEGG: Kyoto Encyclopedia of Genes and Genomes
GO: Gene ontology
SVR: Support vector regression
LASSO: Least absolute shrinkage and selection operator
DEG: Differentially expressed genes
AUROC: Area under the receiver operating characteristic curve
PPI: Protein–protein interaction
