# Supplementary material for: Latent-space embedding of expression data identifies gene signatures from sputum samples of asthmatic patients
Source: BMC Bioinformatics. 2020 Oct 15;21:457. doi: 10.1186/s12859-020-03785-y (PMC7560063; doi:10.1186/s12859-020-03785-y)
Supplement: Supplementary file 1 — Additional file 1. Supplementary Information for the analysis. Table S1–S3 and Fig S1–S13 are included in the file. [file 12859_2020_3785_MOESM1_ESM.docx]

**Table S1.** 50 selected gene probes.

| **Probe** | **Gene** |
| --- | --- |
| 8156228 | CTSL1 |
| 8051066 | MPV17, MPV17 |
| 8028652 | ZFP36 |
| 7952325 | HSPA8 |
| 8179827 | CLIC1 |
| 7948606 | C11orf10 |
| 8101322 | MOP-1 |
| 8138466 | MACC1 |
| 8146216 | VDAC3 |
| 7985166 | IREB2 |
| 8178891 | HLA-DPA1 |
| 8041015 | SLC4A1AP |
| 8156538 | HIATL1 |
| 8125556 | HLA-DPA1 |
| 7989735 | CLPX |
| 7904726 | TXNIP |
| 8115831 | DUSP1 |
| 7978905 | RN7SL2, RN7SL1 |
| 8117800 | HLA-B, HLA-A, HLA-A |
| 8088526 | THOC7 |
| 8046604 | AGPS |
| 7934196 | PSAP |
| 7905571 | S100A9 |
| 7965812 | GNPTAB |
| 8178435 | IER3 |
| 8054611 | LOC541471, LINC00152 |
| 8112033 | ARL15 |
| 8068593 | ETS2 |
| 8114861 | LARS |
| 7953385 | GAPDH |
| 8043363 | LOC541471, LINC00152 |
| 8071649 | LOC96610, BMS1 |
| 7985662 | PDE8A |
| 7933084 | NAMPT |
| 8056201 | RBMS1 |
| 8069541 | SAMSN1, SAMSN1 |
| 8154531 | DENND4C |
| 8177732 | HLA-B, HLA-A |
| 7961142 | OLR1 |
| 8166469 | SAT1 |
| 7954997 | ANO6 |
| 7903507 | FAM102B |
| 7935776 | SCD |
| 7968344 | ALOX5AP |
| 7960865 | SLC2A3 |
| 7963046 | TUBA1B |
| 8131844 | GPNMB |
| 7962085 | IPO8 |
| 8040386 | DDX1 |
| 7981722 | IGHG1, IGHA1, IGHV3-48 |

**Table S2.**Support Vector Regression results (MSE and variance explained).

| **Traits (mean)** | **DEG** | **H.var** | **H.sig** | **top.genes** | **top.genes.selected** |
| --- | --- | --- | --- | --- | --- |
| POST FEV1/FVC  (0.7288) | 0.0168  (-0.147) | 0.0151  (-0.0502) | 0.0138  (0.0556) | 0.0119  (0.1828) | 0.0116  (0.1909) |
| PRE FEV1/FVC  (0.6976) | 0.0169  (-0.0865) | 0.0166  (-0.1414) | 0.0147  (-0.0157) | 0.0122  (0.1426) | 0.0122  (0.1522) |

**Table S3.** LASSO results (MSE and variance explained).

| **Traits (mean)** | **DEG** | **H.var** | **H.sig** | **top.genes** | **top.genes.selected** |
| --- | --- | --- | --- | --- | --- |
| POST FEV1/FVC  (0.7288) | 0.0154  (-0.0864) | 0.0139  (0.0227) | 0.0135  (0.0563) | 0.0128  (0.0569) | 0.0121  (0.134) |
| PRE FEV1/FVC  (0.6976) | 0.0154  (-0.0835) | 0.0141  (0.0004) | 0.0139  (0.0284) | 0.0138  (-0.008) | 0.0130  (0.071) |

**Fig S1.** Convergence of the average training and test loss.

**Fig S2.** Convergence of cross validation with 10 repeats.

**Fig S3.** Selection of number of hidden units based on test error.

** Fig S4.** Demographics of the TEA clusters.

** Fig S5.** Spearman correlation between hidden unit values and TEA cluster labels.

**Fig S6.** GSEA results of two sample hidden units using encoder weights, H26 from the negative set and H38 from the positive set.

**Fig S7.** Gene set enrichment of asthma pathway from KEGG (KEGG_ASTHMA) for H27, H36 and H45

**Fig S8.** GSEA results of two sample hidden units using encoder weights, H26 from the negative set and H38 from the positive set.

** Fig S9.** Correlation between hidden unit values and clinical traits.

**Fig S10.** AUROC of the prediction of asthma severity using different feature sets.

**Fig S11.** Comparison of network centrality between our selected genes and severity-related DEGs.

** Fig S12.** Plot of predicted value v.s true value of pre-treatment FEV1/FVC using LASSO with selected gene expression.

**Fig S13.** Plot of predicted value v.s true value of post-treatment FEV1/FVC using LASSO with selected gene expression.

.
